# Normal Diet within Two Postoperative Days—Realistic or Too Ambitious?

**DOI:** 10.3390/nu9121336

**Published:** 2017-12-08

**Authors:** Fabian Grass, Markus Schäfer, Nicolas Demartines, Martin Hübner

**Affiliations:** Department of Visceral Surgery, Lausanne University Hospital CHUV, Lausanne 1011, Switzerland; fabian.grass@chuv.ch (F.G.); markus.schafer@chuv.ch (M.S.); martin.hubner@chuv.ch (M.H.)

**Keywords:** nutrition, diet, colorectal surgery, risk factors, enhanced recovery

## Abstract

Enhanced Recovery After Surgery (ERAS) protocols advocate early postoperative resumption of normal diet to decrease surgical stress and prevent excessive catabolism. The aim of the present study was to identify reasons for delayed tolerance of normal postoperative diet. This was a retrospective analysis including all consecutive colorectal surgical procedures since May 2011 until May 2017. Data was prospectively recorded by an institutional data manager in a dedicated database. Uni- and multivariate risk factors associated with delayed diet (beyond POD 2) were identified by multiple logistic regression among demographic, surgery- and modifiable pre- and intraoperative ERAS-related items. In a second step, univariate analysis was performed to compare surgical outcomes for patients with early vs. delayed oral intake. The study cohort consisted of 1301 consecutive colorectal ERAS patients. Herein, 691 patients (53%) were able to resume normal diet within two days of surgery according to ERAS protocol, while in 610 patients (47%), a delay in tolerance of normal diet was observed. Male gender was independently correlated to early tolerance (Odds Ratio (OR) 0.66; 95% Confidence Interval (CI) 0.46–0.84, *p* = 0.002), while ASA score ≥ 3 (OR 1.60; 95% CI 1.12–2.28, *p* = 0.010), abdominal drains (OR 1.80; 95% CI 1.10–2.49, *p* = 0.020), right colectomy (OR 1.64; 95% CI 1.08–2.49, *p* = 0.020) and Hartmann reversal (OR 2.61; 95% CI 1.32–5.18, *p* = 0.006) constituted risk factors for delayed tolerance of normal diet. Patients with delayed resumption of normal diet experienced more overall (Clavien grade I–V) (47% vs. 21%, *p* < 0.001) and major (Clavien grade IIIb–V) (11% vs. 4%, *p* < 0.001) complications and had a longer length of stay (9 ± 5 vs. 5 ± 4 days, *p* < 0.001). Over half of patients could not tolerate early enteral realimentation and were at higher risk for postoperative complications. Prophylactic drain placement was the only independent modifiable risk factor for delayed oral intake.

## 1. Introduction

Early resumption of normal solid diet is advocated by Enhanced Recovery After Surgery (ERAS) guidelines to keep the patient in a “close to normal” state through the perioperative period aiming to decrease surgical stress response [1,2,3]. Together with concomitant measures including minimal preoperative fasting and carbohydrate loading, early oral intake is an important part of the multimodal ERAS care strategy that has been consistently associated with decreased morbidity, length of stay and costs [4,5]. While tolerance of early oral feeding was not influenced by postoperative ileus in a randomized controlled trial [6], other studies revealed even accelerated gastrointestinal recovery by early resumption of oral diet [7,8]. Thus, the concept of early oral intake gained even more interest. Combined with forced mobilisation and epidural analgesia, hormonal and metabolic stress could be significantly decreased [9]. In daily practice, however, not all patients return to normal diet as suggested.

The aim of the present study was to assess tolerance of early postoperative oral re-alimentation after colorectal surgery within ERAS care and to identify risk factors associated with delayed return to normal “everyday” diet.

## 2. Materials and Methods

### 2.1. Patients

The study cohort included of all consecutive adult colorectal surgical patients operated between 1 May 2011 and 31 May 2017 at the Department of Visceral Surgery, Lausanne University Hospital (CHUV). All patients were treated within a standardized ERAS pathway [10]. All elective procedures were included. Emergency procedures were included since April 2012 [11]. This study was approved by the Institutional Review Board (Commission cantonale d’éthique de la recherche sur l’être humain CER-VD, # 2017-01991). Written informed consent was obtained from every patient. Patient records and information were anonymized and de-identified prior to further analysis. The study was conducted according to the STROBE criteria and registered under www.researchregistry.com (UIN research registry 3159).

Baseline demographic and surgical information was prospectively recorded in a dedicated ERAS database by two institutional ERAS study nurses and analyzed with the ERAS Interactive Audit System (EIAS). Accuracy of data entry was cross-checked by audit sessions attended by data manager, ERAS nurses, surgeons, and anesthesiologists of the Institutional ERAS team. Age, gender, Body Mass Index (BMI), American Society of Anesthesiologists (ASA) and World Health Organization (WHO) mobility performance scores, and social habits such as smoking or alcohol abuse (use of alcoholic beverages to excess as a regular practice) at the time of the procedure were recorded. Further were assessed preoperative malnutrition, defined as a Nutritional Risk Score ≥ 3 [12], and preoperative daily enteral or parenteral nutritional support. Immunocompromised state due to immunosuppressive medication, preoperative chemo- or radiotherapy and diabetes mellitus was evaluated. The underlying disease was stratified between malignant disease, diverticulitis, inflammatory bowel disease (either Crohn’s disease or ulcerative colitis), functional disorder, or other benign pathologies. Previous abdominal surgery was recorded.

Main surgical procedures were classified as colectomies (left and sigmoidal, right or total), rectal resections including low anterior resection, proctocolectomy, and abdominoperineal resection, and stoma procedures (either Hartmann reversal or loop ileostomy closure). Further recorded surgical items were approach (minimally invasive vs. open), setting (elective vs. emergency, defined as any procedure performed during an unplanned hospital admission), duration, realization of bowel anastomosis or new stoma (either protective or end-stoma), intraoperative blood loss, and length of surgical incision.

### 2.2. Assessment of Compliance to ERAS Items

All perioperative care items of the ERAS protocol [1] were systematically recorded. Nineteen pre-, peri-, and postoperative ERAS care items were assessed and stratified with a cutoff of 70% [13,14] to indicate sufficient overall compliance.

Every individual modifiable pre- and intraoperative care item was further compared between the two groups (fast return to normal diet vs. delayed tolerance of normal diet). These items were: preadmission patient education, preoperative oral carbohydrate drinks, no oral bowel preparation, no preoperative long acting sedative medication, antibiotic prophylaxis, thrombo-prophylaxis, no abdominal drains, postoperative nausea and vomiting (PONV) prophylaxis (droperidol 1 mg, ondansetron 4 mg, bethamethasone 4 mg), hypothermia prevention (active warming by air blanket), intraoperative total fluid administration of <2000 mL, fluid administration guidance, no prophylactic nasogastric tube (NGT), and intraoperative thoracic epidural analgesia (EDA).

### 2.3. Outcomes/Study Endpoints

The primary endpoint was the ability to tolerate normal solid food at postoperative day (POD) 2 according to ERAS guidelines [1] by use of the following definition: ability to eat at least two successive normal meals without vomiting and/or reinsertion of nasogastric tube. Patients needed to eat at least 2/3 of the plate *or* at least the usual amount of food ingested before the operation. Quantity of ingested meal was patient-reported daily in a dedicated diary and cross-checked by the ward nurse or a specialized nutritionist if nutritional follow-up was needed. Of note, patients were further supplemented with oral nutritional supplements if caloric needs were not met by normal diet alone. All patients who did not meet these criteria at POD 2 were defined as patients with delayed tolerance of normal diet. Variables associated with delayed tolerance of normal diet were identified among baseline demographic, surgery-related and modifiable pre- and intraoperative ERAS care items.

Clinical outcome was evaluated until 30 days postoperatively and compared between the two groups (fast return to normal diet vs. delayed normal diet). Overall complications were assessed and classified according to the Clavien classification score [15] as any complication (Clavien I–V) and major complication (Clavien IIIb–V). Further were recorded surgical complications (causal relationship between complication and surgical procedure established), infectious complications, cardiovascular complications (including dysrhythmia and angina pectoris or myocardial infarction) and respiratory complications (including respiratory failure or pneumonia), reoperation rates, and length of hospital stay.

### 2.4. Statistical Analysis

Descriptive statistics for categorical variables were reported as frequency (%), while continuous variables were reported as mean (standard deviation). Chi-square was used for comparison of categorical variables. All statistical tests were two-sided and a level of 0.05 was used to indicate statistical significance. Variables with *p* values ≤ 0.05 were then entered into a multivariate logistic regression (based on a probit regression model) to provide adjusted estimations of the odds ratio (OR). Items with <10% event rate were excluded from analysis. Data analysis was performed with the Statistical Software for the Social Sciences SPSS Advanced Statistics 22 (IBM Software Group, 200 W. Madison St., Chicago, IL, USA).

## 3. Results

### 3.1. Patients

A total of 1301 patients (774 male and 527 female) underwent colorectal surgical procedures during the study period. Baseline characteristics are displayed in Table 1 and surgical details in Table 2. The significant association in the subgroup other surgical procedures was due to disease extent in this subgroup of advanced oncological patients undergoing exclusively palliative resections that could not be assigned to one type of intervention. Delayed tolerance of normal food had thus to be expected in this subgroup, which was not retained for multivariate analysis.

Mean time to tolerate solid food was 2.5 ± 5.3 days for ileostomy closure, 2.9 ± 3.1 days for left colectomy, 3.5 ± 3.4 days for right colectomy, 3.5 ± 4.4 days for rectum procedure, 3.8 ± 3.1 days for total colectomy, and 4.5 ± 5.3 for Hartmann reversal.

### 3.2. ERAS Compliance and Modifiable Pre- and Intraoperative ERAS Items

**Figure 1:**
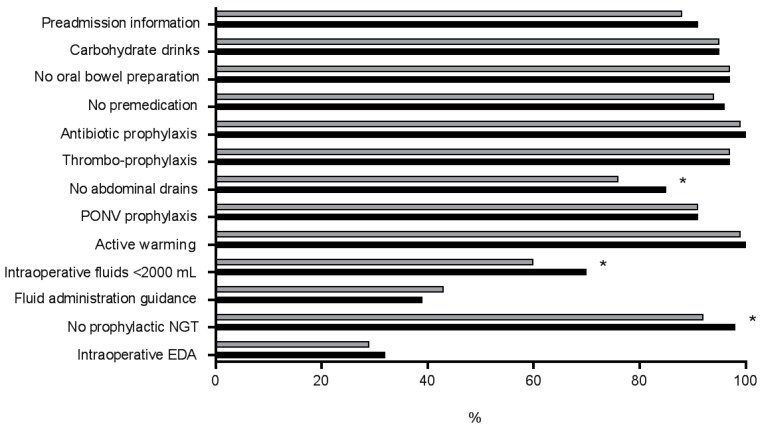
Enhanced Recovery After Surgery (ERAS) compliance. Comparison of compliance to modifiable pre- and intraoperative ERAS-related items among patients with fast return to normal dietary intake (within POD 2, black bars) and patients with delayed tolerance of normal diet beyond POD 2 (grey bars). Premedication = administration of long-acting sedative medication, PONV: postoperative nausea and vomiting, NGT: nasogastric tube; EDA: epidural analgesia. * Indicates statistical significance (*p* < 0.05).

There were 912 patients (70%) who presented an overall compliance to ERAS items of at least 70% with significantly higher percentage for patients with early tolerance of oral intake (73% vs. 67%, *p* = 0.019). Modifiable pre- and intraoperative ERAS items are illustrated in Figure 1.

Differences between the two groups were observed among the following items: No abdominal drains (85% vs. 76%, *p* = 0.002), intraoperative fluid administration of <2000 mL (70% vs. 60%, *p* = 0.004) and no prophylactic NGT (98% vs. 92%, *p* = 0.005).

### 3.3. Factors Associated with Delayed Tolerance of Normal Diet

Univariate demographic and surgical risk factors (*p* < 0.05) associated with delayed tolerance of normal diet are displayed in Table 1 and Table 2.

**Figure 2:**
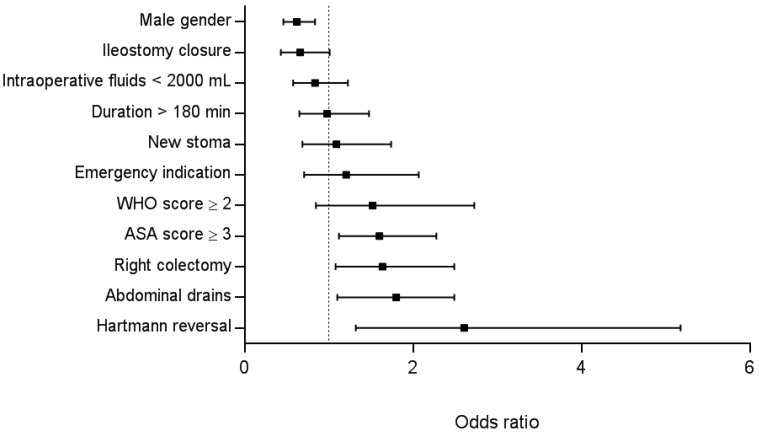
Multivariate analysis. Multivariate analysis of univariate factors with *p* < 0.05 associated with delayed tolerance of normal diet. An Odds ratio of more than 1 increases the risk of delayed normal diet. ASA: American Society of Anaesthesiology; WHO score: World Health Organisation performance score; Odds ratio: 95% Confidence Interval.

Multivariate analysis retained male gender (Odds Ratio (OR) 0.66; 95% Confidence Interval (CI) 0.46–0.84, *p* = 0.002) as protective factor, while ASA score ≥ 3 (OR 1.60; 95% CI 1.12–2.28, *p* = 0.010), abdominal drains (OR 1.80; 95% CI 1.10–2.49, *p* = 0.020), right colectomy (OR 1.64; 95% CI 1.08–2.49, *p* = 0.020) and Hartmann reversal (OR 2.61; 95% CI 1.32–5.18, *p* = 0.006) constituted risk factors for delayed tolerance of normal diet (Figure 2).

### 3.4. Outcome

**Figure 3:**
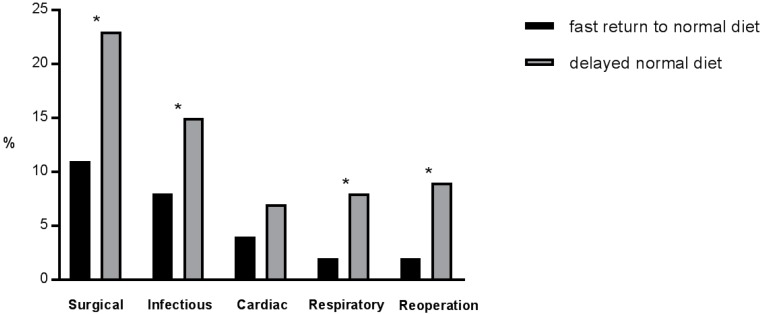
Comparison of surgical, infectious, cardiovascular, and respiratory complications and reoperation rate among patients with fast return to normal dietary intake (within POD 2, black bars) and patients with delayed tolerance of normal diet beyond POD 2 (grey bars). * Indicates statistical significance (*p* < 0.05).

Delayed resumption of normal diet was associated with more overall complications (47% vs. 21% in patients with fast return to normal diet, *p* < 0.001) and major complications (11% vs. 4%, *p* < 0.001). Further, a correlation with surgical (23% vs. 11%, *p* < 0.001), infectious (15% vs. 8%, *p* = 0.001) and respiratory (8% vs. 2%, *p* < 0.001) complications and reoperation rates (9% vs. 2%, *p* < 0.001), but not with cardiovascular complications (7% vs. 4%, *p* = 0.176), was observed (Figure 3).

Mean length of stay was significantly longer for patients with delayed return to normal diet (9 ± 5 vs. 5 ± 4 days, *p* < 0.001).

## 4. Discussion

In the present cohort, representing a typical case mix of colorectal surgical procedures in a high-volume facility, over half of patients were not able to tolerate normal solid food by the end of the second postoperative day, despite care within a standardized ERAS protocol and satisfying overall compliance. The surgical procedure itself had an impact on tolerance of normal food, as demonstrated by lower rates of tolerance after right colectomy and Hartmann reversal. Abdominal drains and prophylactic nasogastric tubes were associated with delayed resumption of normal diet, as were female gender and higher ASA-score as non-modifiable risk factors. Finally, delayed normal food intake was significantly correlated with increased postoperative morbidity and length of stay.

The traditional dogma nihil per os until full functional recovery has been abolished, mainly due to pathophysiologic considerations [16,17]. Surgery comes along with a catabolic response and increased metabolic demands, denoted as perioperative stress response [2,3]. Among simple pre-, peri- and postoperative ERAS measures to counteract this cascade, nutritional considerations play an important role [1]. Insulin sensitivity has been demonstrated to remain near to baseline by applying a combination of preoperative nutritional supplementation, carbohydrate loading and minimal fasting time through the surgical period [3,18]. Embedded in the multimodal and multidisciplinary concept of ERAS, these measures have proven their efficacy by decreasing postoperative morbidity, length of stay and costs [5,19]. In several randomized controlled trials, most patients tolerated early resumption of oral intake after colorectal surgery despite incomplete gastrointestinal functional recovery [6,20]. All studies concluded that there was no reason to withhold early oral intake. These findings were confirmed by recent meta-analyses describing early enteral feeding to decrease length of stay and postoperative complications, similar to the findings of the present study [8,21]. While the evidence to resume normal nutrition within two postoperative days is compelling, it remains unknown how many patients tolerate early nutrition as “real-world” situation. The main finding of the present study is that more than half of patients were not able to respect early realimentation despite high compliance with the ERAS protocol. This goes along with former studies showing similarly low tolerance rates when analyzing compliance to oral nutritional support regimens [22,23].

The present study aimed to identify risk factors associated with delayed tolerance of normal diet through multivariate analysis to limit confounding bias. Three main associations need to be discussed (Figure 2). First, a procedure-specific impaired tolerance was observed. Right-sided colectomy has been associated with delayed functional recovery by our group and others [24,25]. Reasons for this finding however remain unclear. Hartmann reversal represent challenging procedures generally performed by open approach in previously operated patients with complicated disease courses. Thus, the extent of surgery (including extensive adhesiolysis due to previous peritonitis impeding prompt functional recovery) might primarily account for the delay in this subgroup of patients [26]. Loop ileostomy closure was included in the present analysis since these procedures are carried out by colorectal surgeons. Functional recovery was however rarely an issue in these less invasive procedures, leading to rather fast resumption of oral diet in the present cohort [27].

The second finding was the observed better adherence to the ERAS protocol in patients with fast resumption of normal diet. While overall compliance of >70% was unsurprisingly higher in these patients, assessment of individual pre- and intraoperative items was more conclusive. Abdominal drains and NGT were retained as independent risk factors by the present analysis. Both measures are modifiable and well-known to impede functional recovery and should hence be omitted [28]. Thus, even in an established ERAS center and despite ERAS recommendation, “prophylactic drains” are still used.

The significant association of several types of complications with delayed tolerance of normal diet, reflected by increased length of stay, goes along with previous reports [8,21]. This observation however does not allow to draw causal conclusions.

Several limitations of the present study need to be addressed. The study cohort was heterogeneous, although representative of “real clinical life”. Nevertheless, associations have to be interpreted with caution. Numerous factors might impact surgical and functional recovery to finally delay tolerance of normal diet, and straightforward cause–effect patterns cannot be identified in this retrospective study. All available variables in the dataset were analysed. However, other items were not available, potentially leading to residual confounding. Delayed tolerance might also be due to patients’ refusal without an obvious reason, which was not accounted for in the present study. However, two findings of this study need to be emphasized: Full nutrition within two postoperative days was an unachievable goal for more than half of patients in the present cohort. Second, even though a causal explanation could not be established due to the retrospective study design, patients with delayed tolerance of normal diet presented with more complicated recovery, which emphasizes the importance of nutritional follow-up in surgical patients.

## 5. Conclusions

In conclusion, despite standardized care within an enhanced recovery protocol, prompt resumption of a normal “everyday” diet remains a challenge. Adapted protocols with tailored procedure- and patient-specific re-nutrition policies might have to be considered.

## Figures and Tables

**Table 1:** Patients’ characteristics.

	All Patients (*n* = 1301)	Fast Return to Normal Diet (*n* = 691)	Delayed Normal Diet (*n* = 610)	*p*
Age (mean ± SD)	62 ± 15	61 ± 15	62 ± 17	0.367
Gender (m:f)	774:527	433:258	341:269	0.013
BMI (kg/m^2^) (mean ± SD)	25.7± 5.1	25.9 ± 5.0	25.6 ± 5.4	0.394
ASA Group (1-2:3-4)	979:322	542:149	437:173	**0.004**
Smoking (%)	279 (23)	160 (23)	119 (20)	0.110
Alcohol abuse (%)	136 (10)	83 (12)	53 (9)	0.158
Diabetes Mellitus (%)	145 (11)	73 (11)	72 (12)	0.479
Preoperative malnutrition (%)	170 (13)	87 (13)	83 (14)	0.587
Preoperative nutritional treatment (%)	156 (12)	79 (11)	77 (13)	0.509
WHO performance score > 2 (%)	165 (13)	65 (9)	100 (16)	**<0.001**
Preoperative radiotherapy (%)	173 (13)	90 (13)	83 (14)	0.758
Preoperative chemotherapy (%)	181 (14)	97 (14)	84 (14)	0.889
Immunosuppression (%)	140 (11)	67 (10)	73 (12)	0.187
Previous abdominal surgery (%)	590 (45)	324 (47)	266 (44)	0.235
Underlying disease:				
Malignancy	831 (64)	454 (66)	377 (62)	0.144
Diverticulitis	179 (14)	94 (13)	85 (14)	0.863
Inflammatory bowel disease	87 (6)	41 (6)	46 (8)	0.245
Functional disorder	75 (6)	35 (5)	40 (6)	0.249
Other benign condition	129 (10)	67 (10)	62 (10)	0.467

Baseline demographic parameters comparing patients with fast return to normal dietary intake (within postoperative day (POD) 2) (*n* = 691) to patients with delayed tolerance of normal diet beyond POD 2 (*n* = 610). BMI: body mass index; ASA: American Society of Anaesthesiology; WHO: World Health Organisation. Age and BMI are presented as mean ± standard deviation. All others are frequency with percentage. Bold characters indicate significant values (*p* < 0.05).

**Table 2:** Surgical parameter.

	All Patients (*n* = 1301)	Fast Return to Normal Diet (*n* = 691)	Delayed Normal Diet (*n* = 610)	*p*
Surgical procedure:				
Left colectomy (%)	370 (28)	208 (30)	162 (27)	0.157
Right colectomy (%)	252 (19)	117 (17)	135 (22)	**0.018**
Total colectomy (%)	42 (3)	20 (3)	22 (4)	0.468
Rectal procedure (%)	254 (20)	129 (19)	125 (20)	0.408
Hartmann reversal (%)	71 (6)	26 (4)	45 (7)	**0.004**
Ileostomy closure (%)	289 (22)	198 (29)	91 (15)	**<0.001**
Other (%)	23 (2)	4 (1)	19 (3)	**<0.001**
Minimal invasive approach (%)	860 (66)	455 (66)	405 (66)	0.835
Conversion to open approach (%)	81 (9)	40 (9)	41 (10)	0.487
Emergency indication (%)	201 (16)	90 (13)	111 (18)	**0.010**
Operation duration (min) (mean ± SD)	180 ± 90	170 ± 90	190 ± 90	**0.002**
Operation duration > 180 min (%)	472 (36)	227 (33)	245 (40)	**0.006**
New stoma (%)	279 (21)	132 (19)	147 (24)	**0.028**
Bowel anastomosis (%)	1178 (91)	625 (91)	553 (91)	0.899
Hand anastomosis (%)	388 (33)	220 (35)	178 (29)	0.299
Length of incision (cm) (mean ± SD)	12 ± 9	11 ± 9	13 ± 10	**0.004**
Length of incision > 10 cm (%)	566 (44)	284 (41)	282 (46)	0.063

Surgical procedures and parameters comparing patients with fast return to normal dietary intake (within POD 2) (*n* = 691) to patients with delayed tolerance of normal diet beyond POD 2 (*n* = 610). Operation duration, intraoperative blood loss and length of incision are presented as mean ± standard deviation. All others are frequency with percentage. Bold characters indicate significant values (*p* < 0.05).

## References

[1] Gustafsson U.O., Scott M.J., Schwenk W., Demartines N., Roulin D., Francis N., McNaught C.E., MacFie J., Liberman A.S., Soop M. (2013). Guidelines for perioperative care in elective colonic surgery: Enhanced Recovery After Surgery (ERAS^®^) Society recommendations. World J. Surg..

[2] Kehlet H. (1991). The surgical stress response: Should it be prevented?. Can. J. Surg..

[3] Scott M.J., Baldini G., Fearon K.C.H., Feldheiser A., Feldman L.S., Gan T.J., Ljungqvist Q., Lobo D.N., Rockall T.A., Schricker T. (2015). Enhanced Recovery After Surgery (ERAS) for gastrointestinal surgery, part 1: Pathophysiological considerations. Acta Anaesthesiol. Scand..

[4] Gustafsson U.O., Thorell A., Soop M., Ljungqvist O., Nygren J. (2009). Haemoglobin A1c as a predictor of postoperative hyperglycaemia and complications after major colorectal surgery. Br. J. Surg..

[5] Greco M., Capretti G., Beretta L., Gemma M., Pecorelli N., Braga M. (2014). Enhanced recovery program in colorectal surgery: A meta-analysis of randomized controlled trials. World J. Surg..

[6] Han-Geurts I.J., Hop W.C., Kok N.F., Lim A., Brouwer K.J., Jeekel J. (2007). Randomized clinical trial of the impact of early enteral feeding on postoperative ileus and recovery. Br. J. Surg..

[7] Zhang K., Cheng S., Zhu Q., Han Z. (2017). Early versus traditional postoperative oral feeding in patients undergoing elective colorectal surgery: A meta-analysis of safety and efficacy. Zhonghua Wei Chang Wai Ke Za Zhi.

[8] Zhuang C.L., Ye X.Z., Zhang C.J., Dong Q.T., Chen B.C., Yu Z. (2013). Early versus traditional postoperative oral feeding in patients undergoing elective colorectal surgery: A meta-analysis of randomized clinical trials. Dig. Surg..

[9] Brodner G., Van Aken H., Hertle L., Fobker M., Von Eckardstein A., Goeters C., Buerkle H., Harks A., Kehlet H. (2001). Multimodal perioperative management-combining thoracic epidural analgesia, forced mobilization, and oral nutrition-reduces hormonal and metabolic stress and improves convalescence after major urologic surgery. Anesth. Analg..

[10] Roulin D., Donadini A., Gander S., Griesser A.C., Blanc C., Hübner M., Schäfer M., Demartines N. (2013). Cost-effectiveness of the implementation of an enhanced recovery protocol for colorectal surgery. Br. J. Surg..

[11] Roulin D., Blanc C., Muradbegovic M., Hahnloser D., Demartines N., Hübner M. (2014). Enhanced recovery pathway for urgent colectomy. World J. Surg..

[12] Kondrup J., Rasmussen H.H., Hamberg O., Stanga Z. (2003). Nutritional risk screening (NRS 2002): A new method based on an analysis of controlled clinical trials. Clin. Nutr..

[13] Gustafsson U.O., Hausel J., Thorell A., Ljungqvist O., Soop M., Nygren J. (2011). Adherence to the enhanced recovery after surgery protocol and outcomes after colorectal cancer surgery. Arch. Surg..

[14] Jurt J., Slieker J., Frauche P., Addor V., Solà J., Demartines N., Hübner M. (2017). Enhanced Recovery After Surgery: Can We Rely on the Key Factors or Do We Need the Bel Ensemble?. World J. Surg..

[15] Dindo D., Demartines N., Clavien P.A. (2004). Classification of surgical complications: A new proposal with evaluation in a cohort of 6336 patients and results of a survey. Ann. Surg..

[16] Bisgaard T., Kehlet H. (2002). Early oral feeding after elective abdominal surgery—What are the issues?. Nutrition.

[17] Andersen H.K., Lewis S.J., Thomas S. (2006). Early enteral nutrition within 24 h of colorectal surgery versus later commencement of feeding for postoperative complications. Cochrane Database Syst. Rev..

[18] Kiran R.P., Turina M., Hammel J., Fazio V. (2013). The clinical significance of an elevated postoperative glucose value in nondiabetic patients after colorectal surgery: Evidence for the need for tight glucose control?. Ann. Surg..

[19] Feroci F., Lenzi E., Baraghini M., Garzi A., Vannucchi A., Cantafio S., Scatizzi M. (2013). Fast-track colorectal surgery: Protocol adherence influences postoperative outcomes. Int. J. Colorectal. Dis..

[20] Feo C.V., Romanini B., Sortini D., Ragazzi R., Zamboni P., Pansini G.C., Liboni A. (2004). Early oral feeding after colorectal resection: A randomized controlled study. ANZ J. Surg..

[21] Smeets B.J.J., Peters E.G., Horsten E.C.J., Weijs T.J., Rutten H.J.T., Buurman W.A., de Jonge W.J., Luyer M.D.P. (2017). Effect of Early vs. Late Start of Oral Intake on Anastomotic Leakage Following Elective Lower Intestinal Surgery: A Systematic Review. Nutr. Clin. Pract..

[22] Grass F., Bertrand P.C., Schafer M., Ballabeni P., Cerantola Y., Demartines N., Hübner M. (2015). Compliance with preoperative oral nutritional supplements in patients at nutritional risk—Only a question of will?. Eur. Clin. Nutr..

[23] Hiesmayr M., Schindler K., Pernicka E., Schuh C., Schoeniger-Hekele A., Bauer P., Laviano A., Lovell A.D., Mouhieddine M., Schuetz T. (2009). Decreased food intake is a risk factor for mortality in hospitalised patients: The NutritionDay survey 2006. Clin. Nutr..

[24] Kummer A., Hahnloser D., Demartines N., Hubner M. (2017). Comparison of Functional Recovery is Crucial for Implementing ERAS: Reply. World J. Surg..

[25] Vather R., Bissett I.P. (2013). Risk factors for the development of prolonged post-operative ileus following elective colorectal surgery. Int. J. Colorectal. Dis..

[26] Hess G.F., Schafer J., Rosenthal R., Kettelhack C., Oertli D. (2017). Reversal after Hartmann’s procedure in patients with complicated sigmoid diverticulitis. Colorectal. Dis..

[27] Shelygin Y.A., Chernyshov S.V., Rybakov E.G. (2010). Stapled ileostomy closure results in reduction of postoperative morbidity. Tech. Coloproctol..

[28] Nelson R., Tse B., Edwards S. (2005). Systematic review of prophylactic nasogastric decompression after abdominal operations. Br. J. Surg..

